# Atrial Fibrillation Risk Scores as Potential Predictors of Significant Coronary Artery Disease in Chronic Coronary Syndrome: A Novel Diagnostic Approach

**DOI:** 10.3390/life15071134

**Published:** 2025-07-18

**Authors:** Alexandru-Florinel Oancea, Paula Cristina Morariu, Maria Godun, Stefan Dorin Dobreanu, Miron Mihnea, Diana Gabriela Iosep, Ana Maria Buburuz, Ovidiu Mitu, Alexandru Burlacu, Diana-Elena Floria, Raluca Mitea, Andrei Vâță, Daniela Maria Tanase, Antoniu Octavian Petris, Irina-Iuliana Costache-Enache, Mariana Floria

**Affiliations:** 1 Faculty of Medicine, University of Medicine and Pharmacy Grigore T. Popa, 700115 Iasi, Romania; alexandru.oancea@umfiasi.ro (A.-F.O.); morariu.paula-cristina@email.umfiasi.ro (P.C.M.); godun.maria-mihaela@d.umfiasi.ro (M.G.); stefan.dobreanu15@gmail.com (S.D.D.); mironmihnea@yahoo.com.au (M.M.); diana.iosep@umfiasi.ro (D.G.I.); ana-maria.buburuz@umfiasi.ro (A.M.B.); ovidiu.mitu@umfiasi.ro (O.M.); alexandru.burlacu@umfiasi.ro (A.B.); diana-elena.iov@d.umfiasi.ro (D.-E.F.); andrei.vata@umfiasi.ro (A.V.); antoniu.petris@umfiasi.ro (A.O.P.); irina.costache@umfiasi.ro (I.-I.C.-E.); floria.mariana@umfiasi.ro (M.F.); 2 Saint Spiridon Emergency Hospital, 700115 Iasi, Romania; 3 Faculty of Medicine Victor Papilian, University of Lucian Blaga, 550169 Sibiu, Romania; daria.mitea@ulbsibiu.ro; 4 St Parascheva Clinical Hospital of Infectious Diseases, 700116 Iasi, Romania

**Keywords:** risk scores, CHA_2_DS_2_VA, HAS-BLED, C_2_HEST, coronary angiography, clinical prediction tools, chronic coronary syndrome (CCS), atrial fibrillation (AF), coronary artery disease (CAD)

## Abstract

Chronic coronary syndrome (CCS) and atrial fibrillation (AF) are prevalent cardiovascular conditions that share numerous risk factors and pathophysiological mechanisms. While clinical scores commonly used in AF—such as CHA_2_DS_2_VA (which includes congestive heart failure, hypertension, age ≥ 75, diabetes, stroke/TIA, vascular disease, and age 65–74), HAS-BLED (which incorporates hypertension, abnormal renal/liver function, stroke, bleeding history, labile INR, elderly age, and drug/alcohol use), and C_2_HEST (incorporating coronary artery disease, COPD, hypertension, elderly age ≥ 75, systolic heart failure, and thyroid disease)—are traditionally applied to rhythm or bleeding risk prediction, their value in estimating the angiographic severity of coronary artery disease (CAD) remains underexplored. We conducted a prospective, single-center study including 131 patients with suspected stable CAD referred for coronary angiography, stratified according to coronary angiographic findings into two groups: significant coronary stenosis (S-CCS) and non-significant coronary stenosis (N-CCS). At admission, AF-related scores (CHA_2_DS_2_, CHA_2_DS_2_VA, CHA_2_DS_2_VA-HSF, CHA_2_DS_2_VA-RAF, CHA_2_DS_2_VA-LAF, HAS-BLED, C_2_HEST, and HATCH) were calculated. CAD severity was subsequently assessed using the SYNTAX and Gensini scores. Statistical comparisons and Pearson correlation analyses were performed to evaluate the association between clinical risk scores and angiographic findings. Patients in the S-CCS group had significantly higher scores in CHA_2_DS_2_VA (4.09 ± 1.656 vs. 3.20 ± 1.338, *p* = 0.002), HAS-BLED (1.98 ± 0.760 vs. 1.36 ± 0.835, *p* < 0.001), CHA_2_DS_2_VA-HSF (6.00 ± 1.854 vs. 5.26 ± 1.712, *p* = 0.021), and C_2_HEST (3.49 ± 1.501 vs. 2.55 ± 1.279, *p* < 0.001). Multivariate logistic regression identified HAS-BLED and C_2_HEST as independent predictors of significant coronary lesions. A threshold value of HAS-BLED ≥ 1.5 and C_2_HEST ≥ 3.5 demonstrated moderate discriminative ability (AUC = 0.694 and 0.682, respectively), with acceptable sensitivity and specificity. These scores also demonstrated moderate to strong correlations with both Gensini and SYNTAX scores. AF-related clinical scores, especially HAS-BLED and C_2_HEST, may serve as practical and accessible tools for early CAD risk stratification in patients with suspected CCS. Their application in clinical practice may serve as supplementary triage tools to help prioritize patients for further diagnostic evaluation, but they are not intended to replace standard imaging or testing.

## 1. Introduction

Chronic coronary syndrome (CCS) and atrial fibrillation (AF) represent two of the most prevalent cardiovascular conditions worldwide, frequently coexisting in clinical practice and compounding morbidity and mortality risks when present together. Both entities share multiple pathophysiological pathways, including systemic inflammation, endothelial dysfunction, and neurohormonal activation, which contribute to both arrhythmogenic and atherothrombotic processes [1,2,3]. Traditionally, clinical risk scores derived from AF populations, such as CHA_2_DS_2_VA or HAS-BLED, have been utilized primarily for estimating the risk of thromboembolic events or major bleeding in patients requiring anticoagulation [4]. However, increasing evidence suggests that several components of these scores—including age, hypertension, and diabetes mellitus—also represent established risk factors for coronary artery disease (CAD) [5].

Given this shared risk profile, it is plausible that AF-related clinical scores may carry additional value beyond their initial scope. Despite their widespread use, these scores have rarely been investigated as tools for identifying angiographic CAD severity. This represents a critical gap in clinical practice, especially considering the lack of simple, non-invasive methods to predict CAD burden in patients presenting with AF. Traditional CAD risk models often rely on laboratory markers or imaging techniques, which may not always be accessible during early assessment [6].

In this context, repurposing widely used AF-related scores—such as CHA_2_DS_2_VA, HAS-BLED, and C_2_HEST—for the early detection of significant coronary lesions may offer a pragmatic and accessible strategy for cardiovascular risk stratification. Their integration into clinical decision-making pathways could improve triage and resource allocation, particularly in settings with limited access to advanced diagnostics [7]. Subsequently, several modified versions of the classical CHA_2_DS_2_VA score have emerged to improve predictive accuracy in broader cardiovascular contexts. Among these, CHA_2_DS_2_VA-HSF incorporates hyperlipidemia, smoking, and family history to enhance atherosclerotic risk stratification [8]. The CHA_2_DS_2_VA-RAF variant extends this further by integrating renal dysfunction (R) and the type of AF (paroxysmal, persistent, or permanent), accounting for renal contributions to cardiovascular risk. Similarly, the CHA_2_DS_2_VA-LAF version introduces left atrial enlargement (as the acronym L) and the type of AF (paroxysmal, persistent, or permanent), aiming to capture the atrial myopathy and structural cardiac changes associated with both AF progression and adverse cardiovascular outcomes [9]. Furthermore, newer scores such as C_2_HEST and HATCH, originally conceived to predict incident AF in other cardiovascular populations, integrate variables with established links to coronary disease, raising the possibility that their diagnostic utility may extend beyond arrhythmia prediction alone [10].

The ability to non-invasively predict significant CAD using clinical data alone is of particular relevance in patients with AF and suspected CCS, especially given that this population may have atypical or silent ischemic presentations, and the yield of traditional non-invasive testing is often limited [11]. Therefore, identifying simple, accessible clinical tools that could assist in early stratification of coronary risk holds substantial clinical value.

This proof-of-concept study aimed to explore the association between AF-derived clinical scores and the severity of coronary stenosis in a cohort of patients with CCS and/or AF. We hypothesized that risk scores such as CHA_2_DS_2_VA, its extended variants (HSF, RAF, LAF), HAS-BLED, C_2_HEST, and HATCH would differ according to the severity of coronary artery disease, highlighting their potential utility as diagnostic aids in cardiovascular risk stratification. Accordingly, we sought to evaluate the diagnostic utility of selected AF-related clinical scores in predicting significant CAD, as determined by invasive coronary angiography and anatomical scoring systems, such as the Gensini and SYNTAX scores.

## 2. Materials and Methods

### 2.1. Study Design, Patients, and Investigation

We conducted a prospective proof-of-concept study that included 131 consecutively enrolled patients admitted with an indication for coronary angiography between January and June 2024. Eligible patients were over the age of 18, with or without a diagnosis of AF, who presented with signs suggestive of stable CAD. Inclusion criteria encompassed high-risk clinical profiles such as a strong family history of CAD, hypertension, dyslipidemia, diabetes mellitus, obesity, or active smoking, along with either angina symptoms or positive results on non-invasive diagnostic tests (exercise stress test, stress echocardiography, myocardial scintigraphy) or documented coronary stenosis on coronary computed tomography angiography. All participants were required to provide written informed consent and demonstrate the capacity to understand the nature of the study. Although the same patient cohort was used as in our previously published study exploring galectin-3 and pentraxin-3 as biomarkers in S-CCS [2], the current analysis focuses exclusively on the diagnostic value of widely used AF-related clinical risk scores in relation to coronary disease severity as assessed by invasive coronary angiography. In this context, we evaluated the relationship between AF-related scores and both Gensini and SYNTAX scores.

Exclusion criteria included patients under the age of 18, those who did not provide signed informed consent, individuals with acute myocardial infarction, chronic kidney disease with creatinine clearance below 30 mL/min/1.73 m^2^, hemodynamically significant valvular heart disease (greater than mild severity), advanced heart failure (NYHA class III or IV), as well as patients with significant thyroid or psychiatric disorders.

### 2.2. Clinical Evaluation and Data Collection

For all enrolled patients, comprehensive clinical, electrocardiographic (ECG), echocardiographic, and angiographic data were collected. Demographic data included age, sex, and smoking status. We documented comorbidities such as atrial fibrillation (AF), hypertension (HTN), heart failure (HF), diabetes mellitus (DM), chronic kidney disease (CKD) with moderate impairment (creatinine clearance 30–60 mL/min/1.73 m^2^), chronic obstructive pulmonary disease (COPD), aortic plaques, dyslipidemia, peripheral arterial disease (PAD), transient ischemic attack (TIA), and stroke. Chronic medications at baseline were also recorded, including statins, beta-blockers, angiotensin-converting enzyme (ACE) inhibitors, oral anticoagulants, antiplatelet agents, and proton pump inhibitors (PPIs).

Coronary angiography was performed using an Azurion 7 Philips system. Lesion severity was assessed visually, with FFR (fractional flow reserve) or iFR (instantaneous wave-free ratio) used selectively for borderline lesions.

Clinical risk scores specified for AF (CHA_2_DS_2_, CHA_2_DS_2_VA, CHA_2_DS_2_VA-HSF, CHA_2_DS_2_VA-RAF, CHA_2_DS_2_VA-LAF, HAS-BLED, C_2_HEST, and HATCH) were calculated prospectively at the time of admission, based on clinical and historical data routinely collected. Each score was computed by two independent investigators using standardized criteria from established guidelines; any discrepancies were resolved by consensus after reviewing source documentation. Score selection was based on clinical relevance, guideline inclusion, and routine use in AF evaluation. Anatomical and functional coronary scores derived from invasive coronary angiography reflecting CAD severity—Gensini and SYNTAX—were calculated after diagnostic coronary angiography was performed, using the angiographic data obtained. To ensure consistency and transparency in score application, Table 1 summarizes the clinical variables and scoring criteria used for the calculation of each score included in the study.

### 2.3. Statistical Analysis

Statistical analyses were performed using the SPSS software package, version 29.0 (IBM Corp., Armonk, NY, USA). Descriptive statistics were computed for all numerical variables, including mean, standard deviation, median, minimum, and maximum values. For comparisons of quantitative variables between two groups, we employed Student’s t-test, provided that normal distribution was confirmed using the Kolmogorov–Smirnov goodness-of-fit test. When comparing quantitative variables across more than two groups, we applied ANOVA (analysis of variance) if assumptions of normality and homogeneity of variances were satisfied (the latter verified via Levene’s test). In cases where variance homogeneity was violated, we used the Welch ANOVA test, which offers greater robustness. If the assumption of normality was not met, we utilized non-parametric tests: the Mann–Whitney U test for two-group comparisons and the Kruskal–Wallis test for multiple groups. When overall significance was identified in multiple-group comparisons, we conducted post hoc tests to determine specific group differences. For standard ANOVA, the Tukey–Kramer post hoc test was used to account for unequal group sizes. Following Welch’s ANOVA, the Games–Howell post hoc test was applied. For non-parametric Kruskal–Wallis comparisons, we used the Dunn post hoc test with Bonferroni adjustment. For categorical variables, intergroup comparisons were performed using the chi-squared (χ^2^) test. Correlations between continuous variables were evaluated using the Pearson correlation coefficient (r), with corresponding *p*-values and 95% confidence intervals to assess the direction and strength of associations.

A *p*-value < 0.05 was considered statistically significant, while values < 0.01 were considered highly significant. To assess the diagnostic performance of the evaluated clinical scores, receiver operating characteristic (ROC) curve analysis was performed, and the area under the curve (AUC) was calculated and compared for each parameter.

For multivariate analysis, binary logistic regression was conducted using the forward LR method. Variables initially entered into the model included the AF-related risk scores (CHA_2_DS_2_VA, HAS-BLED, CHA_2_DS_2_VA-HSF, C_2_HEST) and relevant clinical confounders such as age, sex, AF status, hypertension, diabetes mellitus, dyslipidemia, and baseline medications (e.g., statins, beta-blockers, anticoagulants). Interaction terms (e.g., HAS-BLED × AF status; C_2_HEST × age ≥ 75) were tested during preliminary modeling but did not reach statistical significance and were excluded from the final model.

To assess potential multicollinearity among predictors, we calculated variance inflation factors (VIFs). All VIF values in the final model were <2.5, indicating no significant collinearity. Model performance was assessed by the Hosmer–Lemeshow goodness-of-fit test, Nagelkerke’s R^2^, classification accuracy, sensitivity, and specificity.

### 2.4. Ethics

This proof-of-concept study was conducted in accordance with the ethical principles outlined in the Declaration of Helsinki, as revised in 2013. Upon admission, all participants provided written informed consent after receiving detailed explanations regarding the study objectives, procedures, and their rights as participants. The study protocol was reviewed and approved by the Ethics Committee of the University of Medicine and Pharmacy “Gr. T. Popa” Iași (Approval No. 352/9 October 2023) and the Ethics Committee of St. Spiridon Emergency Clinical Hospital, Iași (Approval No. 75/11 September 2023).

## 3. Results

### 3.1. Baseline Characteristics

A total of 131 patients were enrolled in the study and stratified into two subgroups based on coronary angiography findings:

- A total of 65 patients were classified as having significant chronic coronary syndrome (S-CCS), defined as ≥70% stenosis in at least one coronary artery (or ≥50% for the left main coronary artery).
- A total of 66 patients were classified as having non-significant chronic coronary syndrome (N-CCS), defined as <70% stenosis in coronary arteries (or <50% for the left main artery). The N-CCS group included individuals with ANOCA (angina with non-obstructive coronary arteries), a condition of increasing clinical interest due to its distinct underlying mechanisms, such as microvascular dysfunction or coronary vasospasm, despite the absence of obstructive coronary lesions [12,13].

Table 2 summarizes the general characteristics, comorbidities, treatment profiles, echocardiographic findings, and laboratory parameters of the study population.

Patients in the S-CCS group demonstrated a significantly higher prevalence of major cardiovascular risk factors, including dyslipidemia, diabetes mellitus, and reduced HDL-cholesterol levels, compared to those in the N-CCS group. However, no significant differences were observed between the groups regarding age, sex, family history of coronary artery disease, smoking status, hypertension, or obesity. A statistically significant difference was noted in cardiac rhythm, with sinus rhythm being more frequent in the S-CCS group (53.8%) compared to the N-CCS group (37.9%, *p* = 0.026). Conversely, atrial fibrillation (AF) was more prevalent in the N-CCS group (62.1%) than in the S-CCS group (46.2%), with a *p*-value of 0.067, suggesting a near-significant trend. The overall prevalence of peripheral arterial disease (PAD) in the cohort was 9.9%, with a significantly higher occurrence in the S-CCS group (15.4%) compared to the N-CCS group (4.5%, *p* = 0.038).

None of the echocardiographic parameters were statistically significantly different in patients with significant versus non-significant coronary lesions.

Regarding laboratory findings, high-density lipoprotein cholesterol (HDLc) was the only lipid marker that significantly differed between groups. Patients with significant coronary lesions exhibited notably significantly lower HDLc levels (mean: 40.88 mg/dL) compared to those with non-significant lesions (mean: 46.06 mg/dL), with a *p*-value of 0.004. This suggests that HDLc may serve as a potentially valuable biomarker in evaluating the extent of CAD.

Treatment regimens varied substantially across the two subgroups. Among patients diagnosed with significant coronary artery disease, 60 underwent percutaneous coronary intervention (PCI) and received dual antiplatelet therapy (DAPT), while the remaining five underwent coronary artery bypass grafting (CABG) and were managed with single antiplatelet therapy (SAPT). Proton pump inhibitors (PPIs) were co-administered with DAPT for gastroprotection, in line with clinical guidelines. Furthermore, the widespread prescription of nitrates and trimetazidine in the significant CAD group underscores adherence to evidence-based medical management. The higher prevalence of lipid-lowering therapy in this group further reflects a strong focus on secondary prevention strategies for high-risk atherosclerotic patients.

### 3.2. Potential Assessment of Coronary Lesion Severity Using AF-Related Risk Scores

To assess the diagnostic potential of AF-related clinical risk scores in predicting significant coronary artery stenosis, we compared multiple scoring systems between patients with significant and non-significant coronary lesions. The findings, summarized in Table 3, illustrate the distribution of these scores—as well as the Gensini score—across patients with N-CCS and those with S-CCS, as determined by coronary angiography.

Unsurprisingly, the Gensini score, a well-validated indicator of coronary lesion burden, was markedly higher in the S-CCS group (mean: 46.83 ± 39.3) compared to the N-CCS group (mean: 5.07 ± 4.96), with a highly significant *p*-value (<0.001). This confirms the expected stratification in coronary severity between the two groups and serves as a reliable foundation for further comparative analysis of clinical risk profiles.

Among the AF-related scores, several showed significant differences between groups, pointing to a potentially meaningful link between AF risk and CAD severity:

- The CHA_2_DS_2_VA score was significantly elevated in the S-CCS group (mean: 4.09 ± 1.656) compared to the N-CCS group (mean: 3.20 ± 1.338, *p* = 0.002). This suggests that patients with more advanced coronary lesions tend to accumulate more systemic vascular risk factors, thereby increasing their AF and thromboembolic risk.
- The HAS-BLED score, used to estimate bleeding risk in anticoagulated AF patients, was also higher among S-CCS patients (1.98 ± 0.760) than in those with N-CCS (1.36 ± 0.835), with a statistically significant *p*-value (<0.001). This may reflect a greater prevalence of comorbidities and pharmacological interventions in the S-CCS group, which complicates antithrombotic management.
- Additionally, the CHA_2_DS_2_VA-HSF variant, incorporating hyperlipidemia, smoking status, and family history of premature CAD, showed a significant elevation in the S-CCS group (mean: 6.00 ± 1.854 vs. 5.26 ± 1.712, *p* = 0.021).

Of particular interest, the C_2_HEST score, developed to predict incident AF in individuals without prior arrhythmia, also differed significantly between the two groups (3.49 ± 1.501 in S-CCS vs. 2.55 ± 1.279 in N-CCS, *p* < 0.001). This reinforces the hypothesis that more extensive coronary atherosclerosis is linked to a higher likelihood of developing AF, possibly via shared mechanisms such as atrial remodeling, inflammation, and myocardial ischemia.

In contrast, other scores—such as CHA_2_DS_2_, CHA_2_DS_2_VA-RAF, CHA_2_DS_2_VA-LAF, and HATCH—did not show statistically significant differences between groups. This may indicate limited sensitivity of these particular models for detecting angiographic CAD severity in this clinical context.

### 3.3. Diagnostic Performance of AF-Related Risk Scores in CCS

To evaluate the diagnostic performance of the AF-related risk scores, we included in a binary logistic regression model the four scores that showed statistically significant differences between patients with and without S-CCS: CHA_2_DS_2_VA, HAS-BLED, CHA_2_DS_2_VA-HSF, and C_2_HEST. The model was built using the Forward LR method. The Hosmer–Lemeshow goodness-of-fit test indicated that the model was viable, as the result was not statistically significant (*p* = 0.232). Initially, the model had a prediction accuracy for S-CCS of 50.4%; after incorporating the selected predictors, the accuracy increased to 71.8%, suggesting that the identified variables contribute meaningfully to diagnostic discrimination. The model explained 31.6% of the variance in the S-CCS diagnosis (Nagelkerke R^2^ = 0.316) and demonstrated a sensitivity of 69.2% and a specificity of 74.2%. Although not exceptional, these performance metrics indicate a reasonable level of clinical utility. The regression model was further tested for multicollinearity. All the included predictors had variance inflation factors (VIFs) between 1.12 and 2.02, confirming the absence of collinearity issues. Interaction terms (HAS-BLED × AF status, C_2_HEST × age, and others) were also tested but did not improve model fit or reach statistical significance and were therefore excluded. These additional steps reinforce the robustness of the final multivariate model.

Among the four tested predictors, HAS-BLED and C_2_HEST scores emerged as statistically significant contributors to the model. Specifically, each one-point increase in the HAS-BLED score was associated with a 2.585-fold increase in the odds of having significant CAD, assuming the other predictors remained constant. Likewise, each one-point increase in the C_2_HEST score was associated with a 1.564-fold increase in the odds of significant stenosis (Table 4).

**Figure 1:**
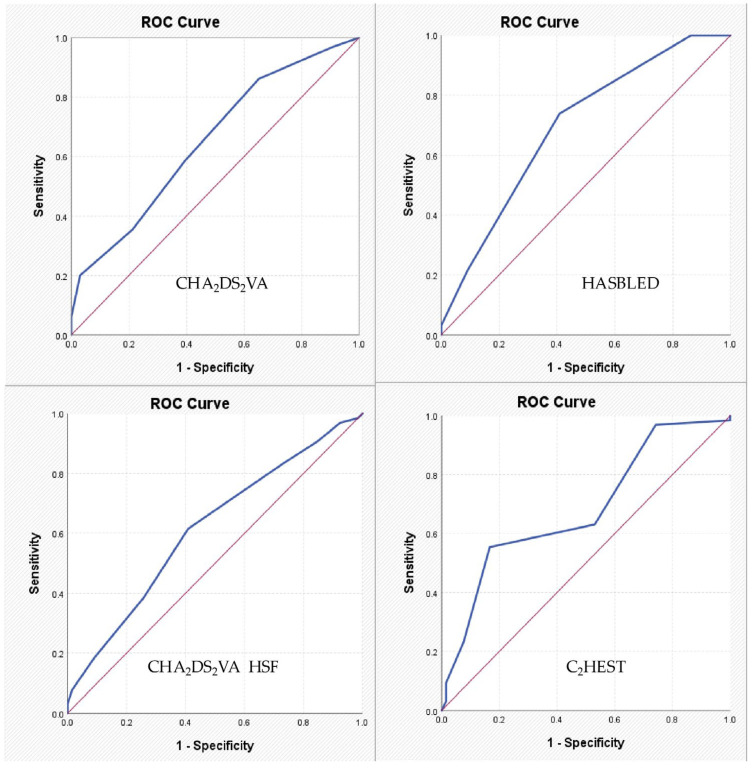
ROC curves expressing the association between specified AF risk scores and the diagnosis of significant chronic coronary syndrome.

For the HAS-BLED score, the identified cut-off value was 1.50. The corresponding AUC coefficient was 0.694, indicating a relatively low discriminative power, with a sensitivity of 73.8% and a specificity of 59.1%. For the CHA_2_DS_2_VA-HSF score, the identified cut-off value was 5.50, with an AUC of 0.615, which also reflects a low discriminative power, with a sensitivity of 61.5% and a specificity of 59.1%. For the C_2_HEST score, the identified cut-off value was 3.50, and the AUC was 0.682, again indicating a relatively low discriminative ability, but with a sensitivity of 55.4% and a specificity of 83.3%. For the remaining parameters, no satisfactory values were recorded (Table 5, Figure 1).

### 3.4. AF-Related Risk Scores: Correlations with Gensini Score

To further explore the potential utility of AF-related clinical scores in estimating the severity of CAD, we assessed the correlation between each score and the Gensini index. The results revealed a statistically significant moderate positive correlation between the HAS-BLED score and the Gensini score (r = 0.368, *p* < 0.0001), as well as between the CHA_2_DS_2_VA score and Gensini (r = 0.273, *p* = 0.0016). These findings suggest that as the clinical burden captured by these scores increases, so does the angiographically quantified severity of CAD. In contrast, the correlations for CHA_2_DS_2_VA-HSF and C_2_HEST scores were weaker and did not reach statistical significance (r = 0.148 and r = 0.154, respectively), with 95% confidence intervals crossing zero. Although the trend remained positive, the lack of statistical significance suggests that these scores may have limited value as standalone predictors of coronary severity in this population. The results are described in Table 6.

Finally, the confidence intervals for HAS-BLED (0.210 to 0.507) and CHA_2_DS_2_VA (0.106 to 0.425) further reinforce the robustness of their associations, as opposed to the broader, zero-inclusive intervals for CHA_2_DS_2_VA-HSF and C_2_HEST. These results underscore that not all AF-related risk scores are equally informative in this context; rather, specific scores such as HAS-BLED and CHA_2_DS_2_VA may reflect overlapping cardiovascular risk factors relevant to both AF and CAD. Consequently, they may serve as practical tools in preliminary risk stratification, particularly when integrated with clinical and imaging data in patients presenting with CCS and/or AF.

### 3.5. AF-Related Risk Scores: Correlations with SYNTAX Score in Patients with S-CCS

Although the SYNTAX score was initially developed to guide revascularization strategies in patients with left main or three-vessel coronary artery disease, we applied it to all patients diagnosed with S-CCS in order to provide a standardized and quantitative assessment of anatomical disease complexity. This broader application was justified by the study’s exploratory aim: to evaluate potential correlations between AF-related clinical risk scores and the angiographic burden of CAD across a range of lesion distributions, not limited to those traditionally associated with SYNTAX. Using this score allowed us to uniformly capture the extent and severity of coronary involvement in S-CCS patients and to explore whether clinical scores might reflect not just the presence, but also the anatomical complexity of CAD. Descriptive statistics for the SYNTAX PCI and CABG scores are presented in Table 7, including mean ± standard deviation and interquartile range (IQR).

Subsequently, we explored whether AF-related clinical risk scores—previously shown to correlate significantly with CAD severity in our cohort—are also associated with the angiographic complexity of coronary disease, as quantified by SYNTAX scoring. To this end, we conducted Pearson correlation analyses between the four clinical scores (CHA_2_DS_2_VA, CHA_2_DS_2_VA-HSF, HAS-BLED, and C_2_HEST) and both SYNTAX PCI and CABG scores. The results are presented in Table 8.

The analysis revealed statistically significant positive correlations between all clinical scores and both SYNTAX PCI and SYNTAX CABG values. Importantly, all *p*-values for the correlations were below the 0.05 threshold, even <0.001, indicating that the observed relationships are highly statistically significant and unlikely to be due to random variation. Furthermore, the calculated 95% confidence intervals for Pearson coefficients confirm the robustness of these associations, as none of them cross zero.

The HAS-BLED score demonstrated the strongest correlation with SYNTAX PCI (r = 0.423, *p* < 0.00001) and SYNTAX CABG (r = 0.430, *p* < 0.00001), suggesting that this score, although originally designed to predict bleeding risk, may also serve as a surrogate marker for CAD severity. This can be explained by the fact that HAS-BLED includes components closely related to vascular dysfunction (e.g., hypertension, renal impairment, age, previous stroke), which are also major contributors to coronary atherosclerosis.

The C_2_HEST score showed a moderate and highly significant correlation with SYNTAX PCI (r = 0.389, *p* < 0.001) and SYNTAX CABG (r = 0.389, *p* < 0.001). As a score intended to predict the development of AF, its association with coronary lesion complexity supports the shared pathophysiologic mechanisms between atrial remodeling and atherosclerosis, including systemic inflammation and endothelial dysfunction.

The CHA_2_DS_2_VA and CHA_2_DS_2_VA-HSF scores also exhibited statistically significant, albeit weaker, correlations with SYNTAX scores (r ≈ 0.29–0.30, *p* < 0.02). Their predictive components—age, hypertension, diabetes, stroke, heart failure, and vascular disease—are directly linked to both AF risk and CAD progression, justifying their moderate association.

Overall, this analysis supports the notion that AF-related clinical risk scores may provide valuable insight into the anatomical severity of CAD in patients with S-CCS. The particularly strong performance of HAS-BLED and C_2_HEST scores suggests their utility may extend beyond their original indications, potentially aiding in risk stratification when angiographic data are not yet available.

## 4. Discussion

The current proof-of-concept study provides novel insight into the potential diagnostic utility of AF-related clinical scores—traditionally used for predicting thromboembolic or rhythm outcomes—in stratifying the severity of CAD in patients with CCS. Among the risk scores evaluated, HAS-BLED and C_2_HEST emerged as independent predictors of significant coronary stenosis, and both demonstrated a statistically significant association with Gensini and SYNTAX scores, validated measures of angiographic CAD burden. These findings support the hypothesis that overlapping pathophysiological substrates between AF and CAD may be captured by multipurpose clinical scores, extending their utility beyond their original scope.

### 4.1. Shared Risk and Pathophysiological Mechanisms

The clinical overlap between AF and CAD is well-established, with shared risk factors including hypertension, diabetes mellitus, obesity, dyslipidemia, and advancing age contributing to both disease entities [12]. Inflammation, oxidative stress, and endothelial dysfunction serve as central mechanisms linking atherogenesis to atrial remodeling, creating a bidirectional relationship between arrhythmogenesis and atherosclerosis. The present proof-of-concept study builds upon this conceptual framework by demonstrating that higher CHA_2_DS_2_VA, CHA_2_DS_2_VA-HSF, C_2_HEST, and HAS-BLED, scores that encapsulate many of these shared risk variables, are associated with a higher prevalence and severity of coronary stenosis [14,15].

### 4.2. CHA_2_DS_2_VA and CHA_2_DS_2_VA-HSF: Beyond Stroke Risk

The CHA_2_DS_2_VA score, the new variant of the well-established CHA_2_DS_2_VASc score, was initially designed to assess thromboembolic risk in patients with AF. However, it incorporates clinical variables such as age, hypertension, diabetes mellitus, and vascular disease—all of which are independently associated with CAD. In our study, this score showed a significant association with the severity of angiographically confirmed CAD, suggesting its potential utility in broader cardiovascular risk stratification beyond AF populations.

Several prior studies have echoed these findings. For instance, Modi et al. investigated a modified version of the score, termed CHA_2_DS_2_VASc-HSF, which includes additional risk factors—hyperlipidemia, smoking, and family history of premature CAD. Their study of 2976 patients undergoing coronary angiography demonstrated that the CHA_2_DS_2_VASc-HSF score had a strong positive correlation with both the presence and severity of CAD, with a statistically significant association (*p* < 0.001). This variant improved the ability to predict significant coronary lesions, underscoring the relevance of incorporating lifestyle and genetic predispositions in cardiovascular risk tools [16].

Moreover, a study by Cetin et al. demonstrated that the CHA_2_DS_2_VASc score positively correlates with the severity and complexity of CAD in patients undergoing coronary angiography. They found that higher CHA_2_DS_2_VASc scores were associated with increased SYNTAX scores, indicating more complex coronary lesions [17]. Furthermore, a study by Tran et al. introduced the CHA_2_DS_2_VASc-HS score, which adds hyperlipidemia and smoking to the original CHA_2_DS_2_VASc components. Their findings revealed a strong correlation between higher CHA_2_DS_2_VASc-HS scores and increased Gensini scores, suggesting that this modified score may be a useful tool for predicting CAD severity [18].

In summary, our findings, supported by the existing literature, indicate that both the CHA_2_DS_2_VA and CHA_2_DS_2_VA-HSF scores, though originally developed for stroke risk in AF management, have broader applicability in assessing CAD severity. These scores, based on readily available clinical parameters, could serve as valuable tools in the early identification and risk stratification of patients with significant CAD.

### 4.3. HAS-BLED Score: A Marker for CAD Risk?

The HAS-BLED score, originally developed to estimate the risk of major bleeding in patients with AF undergoing anticoagulation therapy, includes clinical parameters such as hypertension, abnormal liver or renal function, stroke history, bleeding history, labile INR, age, and concomitant use of drugs or alcohol. While its primary application is in bleeding risk stratification, emerging evidence suggests that the HAS-BLED score may also reflect the overall burden of systemic vascular disease, thereby serving as a potential marker for CAD severity.

In our study, higher HAS-BLED scores were significantly associated with greater CAD severity, as assessed by the Gensini and SYNTAX scores. This finding aligns with previous research indicating that the components of the HAS-BLED score are closely linked to cardiovascular risk factors and outcomes. For instance, a study by Konishi et al. evaluated the predictive value of the HAS-BLED score in patients undergoing PCI with drug-eluting stents. The study found that a high HAS-BLED score (≥3) was independently associated with increased risks of major bleeding and all-cause mortality over a median follow-up of 3.6 years, regardless of the presence of AF. This suggests that the HAS-BLED score captures comorbidities and clinical features that contribute to both bleeding and ischemic risks [19].

Similarly, Castini et al. investigated the utility of the HAS-BLED score for risk stratification in patients with acute coronary syndrome (ACS) without AF. Their study demonstrated that higher HAS-BLED scores were associated with increased in-hospital and post-discharge bleeding events, as well as higher mortality rates. The discriminative performance of the HAS-BLED score for predicting these outcomes was moderate to good, indicating its potential applicability beyond bleeding risk assessment in AF patients [20].

Further evidence supporting the utility of the HAS-BLED score in the stable CAD population is provided by Yildirim et al., who evaluated its performance in predicting hemorrhagic events in patients with chronic CAD receiving antithrombotic therapy. Although the primary focus was on bleeding risk, their findings underscore the applicability of HAS-BLED in this clinical context, demonstrating that it outperformed the CRUSADE score in forecasting major bleeding complications. This is particularly relevant, as many of the components included in HAS-BLED—such as hypertension, renal dysfunction, and prior stroke—are also well-established contributors to CAD progression and adverse outcomes. The study’s emphasis on stable CAD patients highlights that HAS-BLED may not only serve as a bleeding risk tool in anticoagulated populations, but also offer insight into the overall clinical complexity and frailty of CAD patients, further supporting its broader relevance in cardiovascular risk assessment [21].

Taken together, these data support the concept that the HAS-BLED score—though originally designed for assessing bleeding risk in anticoagulated AF patients—may reflect broader vascular risk, including the presence and severity of CAD. Its components overlap substantially with known predictors of adverse cardiovascular events. Therefore, in the setting of CCS, particularly in patients with AF or multiple comorbidities, the HAS-BLED score may serve as a pragmatic and clinically valuable tool not only for anticipating bleeding risk but also for identifying individuals with potentially advanced atherosclerotic disease.

### 4.4. C_2_HEST Score: Predicting More than AF

The C_2_HEST score, originally developed to predict the risk of incident AF, comprises six clinical variables: coronary artery disease (CAD) or chronic obstructive pulmonary disease (COPD) (1 point each), hypertension (1 point), elderly (age ≥ 75 years, 2 points), systolic heart failure (2 points), and thyroid disease (hyperthyroidism, 1 point). While its primary application has been in AF risk stratification, emerging evidence suggests that the C_2_HEST score may also serve as a marker for broader cardiovascular risk, including the severity of CAD [22].

In our study, higher C_2_HEST scores were significantly associated with greater CAD severity, as assessed by the Gensini and SYNTAX scores. This finding aligns with the components of the C_2_HEST score, many of which are established risk factors for atherosclerosis and CAD progression. Supporting this, a study by Li et al. demonstrated that the C_2_HEST score effectively predicted incident AF in a large cohort of post-ischemic stroke patients, with a C-index of 0.734, outperforming other risk scores such as the CHA_2_DS_2_VASc and Framingham risk scores. Although this study focused on AF prediction, the strong performance of the C_2_HEST score underscores its potential utility in assessing overall cardiovascular risk [22].

Moreover, a study by Rola et al. evaluated the utility of the C_2_HEST score in predicting clinical outcomes among hospitalized COVID-19 patients with and without CAD. The study found that higher C_2_HEST scores were associated with increased in-hospital, 3-month, and 6-month mortality rates, particularly in the CAD cohort. Specifically, in the CAD group, in-hospital mortality reached 43.06% in the high-risk C_2_HEST stratum, compared to 26.92% in the non-CAD group. These findings suggest that the C_2_HEST score captures comorbidities and clinical features that contribute to both bleeding and ischemic risks, extending its utility beyond AF prediction to broader cardiovascular risk assessment [23].

These findings collectively indicate that the C_2_HEST score, although originally intended for AF prediction, captures a cluster of clinical features that overlap substantially with the risk profile of patients prone to significant CAD. In our cohort, patients with S-CCS exhibited significantly higher C_2_HEST scores compared to those with non-significant lesions, suggesting that this score may serve as a pragmatic tool for early recognition of more advanced coronary atherosclerosis. As such, the C_2_HEST score could offer additional diagnostic value in identifying individuals at higher risk of severe coronary stenosis, guiding in the selection of patients for invasive coronary angiography.

### 4.5. Clinical Implications, Study Limitations, and Future Directions

The potential repurposing of AF-related clinical scores such as CHA_2_DS_2_VA, CHA_2_DS_2_VA-HSF, HAS-BLED, and C_2_HEST for evaluating the severity of CAD introduces a pragmatic and accessible strategy for early cardiovascular risk stratification. These scores, based on widely available clinical parameters—such as age, hypertension, diabetes, heart failure, and prior vascular disease—offer low-cost, easy-to-use tools that can support decision-making in routine clinical practice. However, given their modest discriminative power (AUCs < 0.70), these scores should be considered as adjunctive rather than definitive tools, and their role is more suitable for preliminary triage rather than diagnostic confirmation.

A practical implication of our findings lies in the potential use of AF-related scores as a rapid, non-invasive screening tool in patients presenting with symptoms suggestive of CAD. In emergency or outpatient settings where immediate access to advanced imaging is limited, clinical scores like HAS-BLED or C_2_HEST could help identify high-risk patients who warrant early referral for invasive coronary assessment. Their simplicity and reliance on routinely collected clinical parameters allow for immediate bedside application without the need for additional laboratory or imaging data. This could streamline diagnostic pathways, reduce unnecessary testing, and optimize resource allocation.

Compared to traditional CAD risk estimation tools such as the Framingham risk score, ASCVD risk calculator, or the SCORE system—which rely primarily on lipid levels, smoking status, and long-term event prediction—AF-derived scores offer a distinct perspective focused on the cumulative burden of systemic comorbidities. While conventional models remain essential for long-term cardiovascular risk stratification in the general population, they may be less informative in acute or atypical presentations, particularly in patients with AF, where arrhythmia-related factors may obscure ischemic symptoms. In contrast, AF-related scores such as HAS-BLED and C_2_HEST capture overlapping clinical variables—age, hypertension, heart failure, and vascular disease—that may indirectly reflect coronary disease burden. Nevertheless, their diagnostic accuracy remains modest, and these scores should be viewed as complementary triage tools rather than replacements for established risk models or diagnostic imaging. Future studies should evaluate whether integrating AF-derived and traditional CAD scores could improve diagnostic precision in selected patient subsets.

It is also important to consider whether the performance of these repurposed scores may vary across different subgroups, such as sex, ethnicity, or comorbidity profiles. For instance, younger patients with non-valvular AF or those without overt cardiovascular symptoms may have deceptively low scores despite harboring significant coronary lesions. Similarly, sex-specific pathophysiological mechanisms could affect score sensitivity, given that women often present with atypical symptoms and microvascular disease. Although our study did not include stratified analyses, these factors should be addressed in future multicenter trials to refine risk-stratification tools across diverse populations.

Several limitations of this study should be acknowledged. First, it was conducted in a single tertiary center, which may limit the generalizability of the findings. Differences in patient demographics, clinical practice patterns, and resource availability across institutions could influence the performance and applicability of these clinical scores in other settings. Second, the sample size—although adequate for a proof-of-concept analysis—was relatively modest (n = 131), potentially limiting the statistical power to detect subtle or borderline significant associations. Third, the severity of coronary stenoses was assessed primarily through visual estimation by experienced operators; although common in clinical practice, this approach is inherently subject to interobserver variability. Fourth, while AF-derived scores demonstrated statistically significant associations with CAD severity, they were not specifically developed for this purpose. Their predictive accuracy in detecting coronary atherosclerosis is inherently constrained by their original design, focused on rhythm and bleeding risk. Consequently, these scores should be viewed as adjunctive, hypothesis-generating tools that may support—but not replace—established diagnostic modalities such as coronary CT angiography or invasive coronary angiography. Finally, the study did not include longitudinal follow-up or clinical outcome tracking, precluding any conclusions regarding prognostic endpoints such as myocardial infarction, revascularization, hospital readmissions, or cardiovascular mortality (MACE).

Future research should focus on validating these findings in larger, multicenter populations, with stratification by age, sex, and clinical presentation. Additionally, developing hybrid risk models that combine clinical scores with imaging and biomarker data may enhance diagnostic precision and risk stratification in patients with suspected CAD.

## 5. Conclusions

Although the present proof-of-concept study highlights potential associations between AF-related clinical scores and CAD severity, these findings should be interpreted with caution. Given the modest sample size, single-center design, and absence of long-term outcome data, the diagnostic utility of these scores remains hypothesis-generating. Further validation in larger, multicenter cohorts is essential before any clinical application can be recommended.

Within this preliminary framework, our results suggest that clinical risk scores primarily developed for AF management—particularly HAS-BLED and C_2_HEST—may reflect angiographic CAD severity, as evidenced by their associations with the Gensini and SYNTAX scores. While promising, these observations must be confirmed in broader populations before drawing firm conclusions.

Given their simplicity, low cost, and broad clinical familiarity, such scores could potentially serve as a rapid triage tool to identify patients with suspected CCS who may benefit from early invasive evaluation. For example, patients with suspected CCS presenting to primary care or emergency departments could be preliminarily stratified based on these scores to prioritize referrals for further testing, such as coronary CT angiography or invasive angiography. However, additional multicenter studies with larger cohorts and longitudinal outcome data are necessary to determine their true diagnostic and prognostic value in the context of CAD.

## Figures and Tables

**Table 1:** Components of the clinical and angiographic risk scores used in the study.

Score	Included Variables	Scoring Details
CHA_2_DS_2_	congestive heart failure, hypertension, Age ≥ 75, diabetes, stroke/TIA	1 point each; age ≥ 75 and stroke/TIA = 2 points
CHA_2_DS_2_VA	CHA_2_DS_2_ + vascular disease (prior MI, PAD, aortic plaque), Age 65–74	vascular disease = 1 point; age 65–74 = 1 point
CHA_2_DS_2_VA-HSF	CHA_2_DS_2_VA + hyperlipidemia, smoking, family history of premature CAD	1 point each for H, S, F
CHA_2_DS_2_VA-RAF	CHA_2_DS_2_VA + renal dysfunction (eGFR < 60), AF type	renal dysfunction = 1 point; AF: paroxysmal = 1 point, persistent = 2 points, permanent = 3 points
CHA_2_DS_2_VA-LAF	CHA_2_DS_2_VA + left atrial enlargement, AF type	LA enlargement = 1 point; AF: paroxysmal = 1 point, persistent = 2 points, permanent = 3 points
HAS-BLED	hypertension, age > 65, stroke, bleeding, labile INR, abnormal liver or renal function, antiplatelets, alcohol use	1 point for each component
C_2_HEST	coronary artery disease, COPD, hypertension, Age ≥ 75, systolic heart failure, thyroid disease	CAD = 1 point; COPD = 1 point; HTN = 1 point; age ≥ 75 = 2 points; HF = 2 points; thyroid disease = 1 point
HATCH	hypertension, age ≥ 75, TIA/stroke, COPD, heart failure	HTN = 1 point; age ≥ 75 = 1 point; TIA/stroke = 2 points; COPD = 1 point; HF = 2 points
SYNTAX II PCI and CABG	coronary anatomy + clinical variables (age, sex, LVEF, eGFR, COPD, PAD)	calculated using official SYNTAX score online calculator (https://syntaxscore.org, accessed on 1 April 2025)
Gensini	Angiographic coronary stenosis and location	stenosis severity (1–32 points) × lesion-specific weighting factor

AF—atrial fibrillation; CABG—coronary artery bypass grafting; CAD—coronary artery disease; COPD—chronic obstructive pulmonary disease; eGFR—estimated glomerular filtration rate; HF—heart failure; HSF—hyperlipidemia, smoking, family history; HTN—hypertension; INR—international normalized ratio; LA—left atrium; LAF—left atrial enlargement, AF type; MI—myocardial infarction; PAD—peripheral arterial disease; PCI—percutaneous coronary intervention; RAF—renal dysfunction, AF type; SYNTAX—synergy between PCI with taxus and cardiac surgery; TIA—transient ischemic attack.

**Table 2:** Selected clinical, echocardiographic, and therapeutic characteristics of the study population by CAD severity.

Parameter	Overall (131 Patients)	N-CCS (66 Patients)	S-CCS (65 Patients)	*p*-Value
**Clinical parameters**				
Age (years)	66.46 ± 8.709	65.56 ± 9.806	67.37 ± 7.396	0.236
Male gender (%)	79 (60.3)	38 (57.6)	41 (63.1)	0.520
Smoking (%)	76 (58.0)	37 (56.1)	39 (60.0)	0.648
**Comorbidites**				
Family history of ischemic coronary disease (%)	77 (58.8)	39 (59.1)	38 (58.5)	0.942
T2DM (%)	47 (35.9)	19 (28.8)	28 (43.1)	0.088
Arterial hypertension (%)	123 (93.9)	60 (90.9)	63 (96.9)	0.274
Obesity (%)	101 (77.1)	54 (81.8)	47 (72.3)	0.195
Dyslipidemia (%)	121 (92.4)	57 (86.4)	64 (98.5)	0.017 *
PAD (%)	13 (9.9)	3 (4.5)	10 (15.4)	0.038 *
CKD (%)	20 (15.3)	9 (13.6)	11 (16.9)	0.601
COPD (%)	16 (12.2)	6 (9.1)	10 (15.4)	0.217
Sinusal rhythm	60 (45.8)	25 (37.9)	35 (53.8)	0.026 *
AF (%)	71 (54.2)	41 (62.1)	30 (46.2)	0.067
Prior stroke/TIA (%)	16 (12.2)	10 (15.2)	6 (9.2)	0.301
**Echocardiography parameters**				
LVDD (mm)	51.21 ± 7.081	51.45 ± 7.506	50.97 ± 6.671	0.982
IVS (mm)	11.21 ± 1.663	11.32 ± 1.824	11.09 ± 1.487	0.562
LVPW (mm)	10.96 ± 1.459	10.94 ± 1.558	10.98 ± 1.364	0.776
LVEDV (mL)	136.54 ± 48.592	138.86 ± 55.791	134.18 ± 40.294	0.876
LVESV (mL)	72.92 ± 38.741	76.39 ± 47.535	69.39 ± 26.993	0.976
LVEF (%)	48.91% ± 11.4%	48.27 ± 12.484	49.57 ± 10.352	0.833
LA indexed volume (mL/m^2^)	38.93 ± 14.292	41.16 ± 16.422	36.67 ± 11.434	0.098
RA indexed volume (mL/m^2^)	31.02 ± 11.715	32.39 ± 13.493	29.63 ± 9.486	0.349
**Biological parameters**				
Glycaemia (mg/dL)	118.34 ± 43.715	115.50 ± 36.457	121.23 ± 50.149	0.406
LDLc (mg/dL)	95.36 ± 37.081	97.95 ± 38.839	92.72 ± 35.311	0.485
HDLc (mg/dL)	43.49 ± 11.385	46.06 ± 11.395	40.88 ± 10.848	0.004 *
Total cholesterol (mg/dL)	158.16 ± 43.588	163.35 ± 43.563	152.89 ± 43.312	0.236
Triglycerides (mg/dL)	120.63 ± 60.578	115.67 ± 53.659	125.68 ± 66.922	0.443
AST (UI/L)	26.08 ± 15.414	27.64 ± 19.584	24.51 ± 9.384	0.750
ALT (UI/L)	25.98 ± 13.413	27.12 ± 16.310	24.82 ± 9.619	0.894
GGT (UI/L)	43.51 ± 43.789	48.92 ± 52.842	38.02 ± 31.593	0.099
Uric acid (mg/dL)	6.14 ± 1.577	6.37 ± 1.611	5.90 ± 1.518	0.089
Creatinine (mg/dL)	0.97 ± 0.284	0.93 ± 0.263	1.00 ± 0.301	0.101
GFR mL/min/1.73 m^2^	77.88 ± 21.050	80.62 ± 22.311	75.09 ± 19.465	0.075
**Treatment**				
ACEI/ARA-II (%)	118 (90.1)	57 (86.4)	61 (93.8)	0.152
BB (%)	107 (81.7)	54 (81.8)	53 (81.5)	0.967
Nitrates (%)	92 (70.2)	27 (40.9)	65 (100.0)	<0.001 *
Trimetazidine (%)	77 (58.8)	12 (18.2)	65 (100.0)	<0.001 *
Lipid-lowering drugs (%)	121 (92.4)	57 (86.4)	64 (98.5)	0.017 *
PPIs (%)	69 (52.6)	9 (13.6)	60 (92.3)	<0.001 *
DAPT (%)	60 (45.8)	0 (0)	60 (92.3)	<0.001 *
SAPT (%)	28 (21.4)	28 (42.4)	0 (0)	<0.001 *
Anticoagulation (%)	71 (54.2)	41 (62.1)	30 (46.2)	0.067
Antiarrhythmics (%)	20 (15.3)	10 (15.2)	10 (15.4)	0.970

* Statistical significance (*p* < 0.05); ACEI—angiotensin-converting enzyme inhibitors; AF—atrial fibrillation; ALT—alanine aminotransferase; ARA-II—angiotensin II receptor antagonists; AST—aspartate transferase; BB—beta-blockers; CKD—chronic kidney disease; COPD—chronic obstructive pulmonary disease; DAPT—dual antiplatelet therapy; GFR—glomerular filtration rate; GGT—gamma-glutamyl transferase; HDLc—high-density lipoprotein-cholesterol; IVS—interventricular septum; LA—left atrium; LDLc—low-density lipoprotein-cholesterol; LVEF—left ventricle ejection fraction; LVDD—left ventricle diastolic diameter; LVEDV—left ventricle end-diastolic volume; LVESV—left ventricle end-systolic volume; LVPW—left ventricle posterior (inferolateral) wall; N-CCS—nonsignificant chronic coronary syndrome; PAD—peripheral artery disease; PPIs—proton pump inhibitors; RA—right atrium; SAPT—single antiplatelet therapy; S-CCS—significant chronic coronary syndrome; TIA—transient ischemic attack; T2DM—type 2 diabetes mellitus.

**Table 3:** Comparison of AF risk scores and Gensini score between patients with significant and non-significant coronary artery stenosis.

Parameter	Group	Mean ± SD	Median (IQR: 25–75)	*p* Value
CHA_2_DS_2_	N-CCS	2.35 ± 1.234	2.00 (1–7)	0.991
	S-CCS	2.42 ± 1.446	2.00 (0–7)	
CHA_2_DS_2_VA	N-CCS	3.20 ± 1.338	3.00 (1–6)	0.002 **
	S-CCS	4.09 ± 1.656	4.00 (1–8)	
HAS-BLED	N-CCS	1.36 ± 0.835	1.00 (0–3)	<0.001 **
	S-CCS	1.98 ± 0.760	2.00 (1–4)	
CHA_2_DS_2_VA-HSF	N-CCS	5.26 ± 1.712	5.00 (1–9)	0.021 *
	S-CCS	6.00 ± 1.854	6.00 (1–11)	
CHA_2_DS_2_VA-RAF	N-CCS	4.91 ± 2.510	5.00 (0–11)	0.562
	S-CCS	5.22 ± 2.342	5.00 (1–11)	
CHA_2_DS_2_VA-LAF	N-CCS	5.35 ± 2.533	6.00 (0–11)	0.645
	S-CCS	5.20 ± 2.538	5.00 (1–11)	
C_2_HEST	N-CCS	2.55 ± 1.279	3.00 (1–7)	<0.001 **
	S-CCS	3.49 ± 1.501	4.00 (0–7)	
HATCH	N-CCS	2.55 ± 1.338	2.50 (1–6)	0.821
	S-CCS	2.49 ± 1.470	3.00 (0–7)	
Gensini Score	N-CCS	5.07 ± 4.96	3.00 (1–27)	<0.001 **
	S-CCS	46.83 ± 39.3	35.00 (4–200)	

* *p*-values calculated using Mann–Whitney U test. Statistical significance: *p* < 0.05 (*), *p* < 0.01 (**); N-CCS: nonsignificant chronic coronary syndrome; S-CCS: significant chronic coronary syndrome.

**Table 4:** Multivariate analysis: HAS-BLED and C_2_HEST potential predictors of S-CCS.

Test Result Variable (s)	B	S.E.	Wald	df	Sig.	Exp(B) = OR	95% C.I. for EXP(B)	
							Lower	Upper
HAS-BLED	0.950	0.279	11.569	1	<0.001 **	2.585	1.495	4.468
C_2_HEST	0.447	0.158	8.040	1	0.005 **	1.564	1.148	2.131
Constant	−1.069	0.772	1.918	1	0.166	0.343		

Statistical significance: *p* < 0.01 (**); S-CCS: significant chronic coronary syndrome.

**Table 5:** Detailed AUC, cut-off value, sensitivity, and specificity for specified parameters.

Test Result Variable (s)	Area Under the Curve				Cut-Off Value	Sensibility	Specificity
	Area	*p*-Value	Asymptotic 95% Confidence Interval				
			Lower Bound	Upper Bound			
CHA_2_DS_2_VA	0.651	0.003 **	0.558	0.744	2.50	0.862	0.348
HAS-BLED	0.694	0.000 **	0.605	0.784	1.50	0.738	0.591
CHA_2_DS_2_VA-HSF	0.615	0.024 *	0.519	0.711	5.50	0.615	0.591
C_2_HEST	0.682	0.000 **	0.591	0.774	3.50	0.554	0.833

Statistical significance: *p* < 0.05 (*), *p* < 0.01 (**).

**Table 6:** Pearson correlation between AF-related risk scores and Gensini score.

Confidence Intervals				
Parameters	Pearson Correlation r	*p*-Value	95% Confidence Interval	
			Lower	Upper
CHA_2_DS_2_VA and Gensini	0.273	0.0016 *	0.106	0.425
HAS-BLED and Gensini	0.368	<0.0001 **	0.210	0.507
CHA_2_DS_2_VA-HSF and Gensini	0.148	0.0911	−0.024	0.312
C_2_HEST and Gensini	0.154	0.0786	−0.018	0.317

Statistical significance: *p* < 0.05 (*), *p* < 0.01 (**).

**Table 7:** Descriptive statistics for SYNTAX scores in patients with S-CCS.

Parameter	Group	Mean ± SD	Median (IQR: 25–75)
SYNTAX PCI	S-CCS	33.52 ± 10.78	30.70 (25.50–40.00)
SYNTAX CABG	S-CCS	30.67 ± 10.48	28.80 (22.40–37.30)

S-CCS: significant chronic coronary syndrome; SYNTAX—synergy between PCI with Taxus and cardiac surgery.

**Table 8:** Pearson correlation between AF-related risk scores and SYNTAX scores in patients with S-CCS.

Confidence Intervals				95% Confidence Interval	
AF Score	SYNTAX Type	Pearson Correlation r	*p*-Value	Lower	Upper
CHA_2_DS_2_VA	SYNTAX PCI	0.535	0.0 **	0.335	0.689
CHA_2_DS_2_VA	SYNTAX CABG	0.39	0.0013 **	0.161	0.579
CHA_2_DS_2_VA-HSF	SYNTAX PCI	0.402	0.0009 **	0.175	0.588
CHA_2_DS_2_VA-HSF	SYNTAX CABG	0.419	0.0005 **	0.195	0.601
HAS-BLED	SYNTAX PCI	0.497	0.0 **	0.288	0.661
HAS-BLED	SYNTAX CABG	0.511	0.0 **	0.305	0.671
C_2_HEST	SYNTAX PCI	0.593	0.0 **	0.408	0.731
C_2_HEST	SYNTAX CABG	0.616	0.0 **	0.438	0.748

Statistical significance: *p* < 0.01 (**).

## Data Availability

All data presented in this study are available within the article. The first author has all the data used in this study.
